# Mitochondrial Dysfunction: A Basic Mechanism in Inflammation-Related Non-Communicable Diseases and Therapeutic Opportunities

**DOI:** 10.1155/2013/135698

**Published:** 2013-02-28

**Authors:** Anna Hernández-Aguilera, Anna Rull, Esther Rodríguez-Gallego, Marta Riera-Borrull, Fedra Luciano-Mateo, Jordi Camps, Javier A. Menéndez, Jorge Joven

**Affiliations:** ^1^Unitat de Recerca Biomèdica, Hospital Universitari Sant Joan, Institut d'Investigació Sanitària Pere Virgili, Universitat Rovira i Virgili, carrer Sant Llorenç 21, 43201 Reus, Spain; ^2^Catalan Institute of Oncology and Girona Biomedical Research Institute, Avda de Francia s/n, 1707 Girona, Spain

## Abstract

Obesity is not necessarily a predisposing factor for disease. It is the handling of fat and/or excessive energy intake that encompasses the linkage of inflammation, oxidation, and metabolism to the deleterious effects associated with the continuous excess of food ingestion. The roles of cytokines and insulin resistance in excessive energy intake have been studied extensively. Tobacco use and obesity accompanied by an unhealthy diet and physical inactivity are the main factors that underlie noncommunicable diseases. The implication is that the management of energy or food intake, which is the main role of mitochondria, is involved in the most common diseases. In this study, we highlight the importance of mitochondrial dysfunction in the mutual relationships between causative conditions. Mitochondria are highly dynamic organelles that fuse and divide in response to environmental stimuli, developmental status, and energy requirements. These organelles act to supply the cell with ATP and to synthesise key molecules in the processes of inflammation, oxidation, and metabolism. Therefore, energy sensors and management effectors are determinants in the course and development of diseases. Regulating mitochondrial function may require a multifaceted approach that includes drugs and plant-derived phenolic compounds with antioxidant and anti-inflammatory activities that improve mitochondrial biogenesis and act to modulate the AMPK/mTOR pathway.

## 1. Background

The burden of noncommunicable diseases is increasing as such diseases are now responsible for more than three in five deaths worldwide. Atherosclerosis and cancer, in which tobacco use and excessive energy intake are determining factors, are the most frequently occurring of these diseases and are potentially preventable [1, 2]. Obesity and associated metabolic disturbances, which have been increasing worldwide in recent years, are the main factors that underlie noncommunicable diseases and are the consequences of unhealthy diets and physical inactivity [3]. Approximately 10–20% of patients with severe obesity, defined as a body mass index (BMI) > 40, present with no other metabolic complications. These patients are referred to by the oxymoronic designation of “metabolically healthy” obese [4–7]. Such a designation implies that most obese patients are not “metabolically healthy. ” Hence, risk factors for the appearance of noncommunicable diseases have emerged. The reasons for these two phenotypes are unknown; the phenotypes might represent different transitions on a disease timeline, and different levels of either chronic inflammation or insulin resistance are likely contributors. Other contributors include gradual differences in glucose tolerance, inflammatory responses, adipose tissue distribution, patterns of adipokine secretion, and age. 

Emerging obesogenic factors are likely to present with significant differences in the elderly, and consequently the prevalence of obesity is expected to increase with increasing age. Therefore, it is likely not coincidental that most co-morbidity associated with obesity and hence with noncommunicable diseases correlates with aging; the processes may share basic mechanisms, particularly mitochondrial age within an individual [7]. Of note, the prevalence of obesity is lower in people over 70 years of age, an effect attributed to the selective mortality of middle-aged people [8]. 

Current recommendations to decrease food intake and increase physical exercise do result in metabolic improvements, but such lifestyle changes are rarely sustained, despite strong motivation. However, several communities have undertaken initiatives to prevent noncommunicable diseases, and the lessons learned from the implementation of such initiatives should be examined further [9]. The active manipulation of energy sensors and effectors might be a possible alternative therapeutic procedure. Our aim is to provide a succinct review of the scarce and disseminated data that link mitochondrial dysfunction to the pathogenesis of energy-related complications and to discuss a possible multifaceted therapeutic approach. 

## 2. Food Availability Links Mitochondrial Dysfunction and the Vicious Cycle of Oxidative Stress and Inflammation

**Figure 1:**
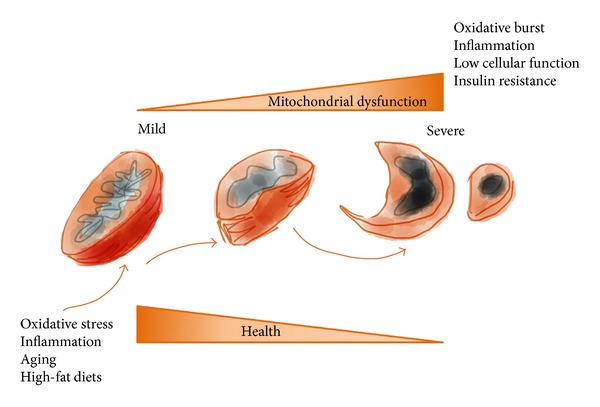
Mutations in mitochondrial DNA are accompanied by different disease-suggestive phenotypes (myopathies, neuropathies, diabetes, and signs of reduced lifespan and premature aging). Severe mitochondrial dysfunction triggers a high level of oxidative and inflammatory damage, impairs tissue function, and promotes age-related diseases.

Mitochondrial defects, systemic inflammation, and oxidative stress are at the root of most noncommunicable diseases such as cancer, atherosclerosis, Parkinson's disease, Alzheimer's disease, other neurodegenerative diseases, heart and lung disturbances, diabetes, obesity, and autoimmune diseases [10–16]. Obesity and obesity-related complications as well as impairment of mitochondrial function, which is required for normal metabolism and health (Figure 1), are universally associated with these conditions. The exact mechanisms that associate mitochondrial dysfunction, obesity, and aging with metabolic syndrome remain a topic of debate [17–22]. 

**Figure 2:**
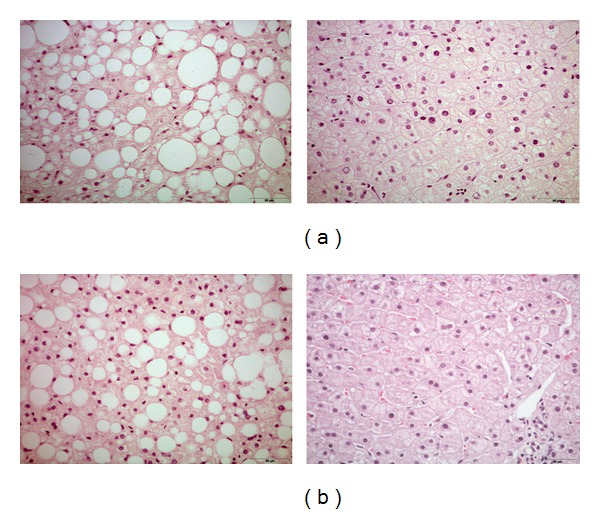
Clinically, it is evident that, in severe obesity, (a) the presence of liver steatosis may vary from more than 80% to less than 5% of patients. Conversely, in most obese patients with some degree of liver steatosis (b), this condition disappeared in a relatively brief period of time after significant weight loss due to bariatric surgery.

Body weight is controlled by molecular messengers that regulate energy status in a limited number of susceptible tissues, including the liver, adipose tissue, skeletal muscles, pancreas, and the hypothalamus [7, 23]. Mouse models of diet-induced obesity have revealed important morphological and molecular differences with respect to humans, particularly those related to the development of fatty liver (NAFLD: nonalcoholic fatty liver disease) or nonalcoholic steatohepatitis (NASH) [24–30] (Figure 2). High expectations for a human therapy after the generation of leptin-deficient animals (Ob/Ob) were countered by the determination that leptin is not a therapeutic option in humans [28]. 

Endoplasmic reticulum (ER) and mitochondrial stress, with the consequent oxidative stress, are immediate consequences of attempts to store excess food energy [23, 29]. Under normal weight conditions, adipose tissue-derived adipokines maintain the homeostasis of glucose and lipid metabolism; however, in obese conditions, the dysregulated production of adipokines favours the development of metabolic syndrome and related complications, particularly the accumulation of triglycerides in nonadipose organs that are not designed to store energy [19]. Other adipokines may cause inflammation and oxidative stress [31], but unknown factors are involved because interventions to ameliorate insulin resistance do not lead uniformly to clinical improvement [32]. It is of paramount importance to understand the mechanisms that disrupt ER homeostasis and lead to the activation of the unfolded protein response and mitochondrial defects in metabolic diseases in order to correctly manage noncommunicable diseases [33]. 

**Figure 3:**
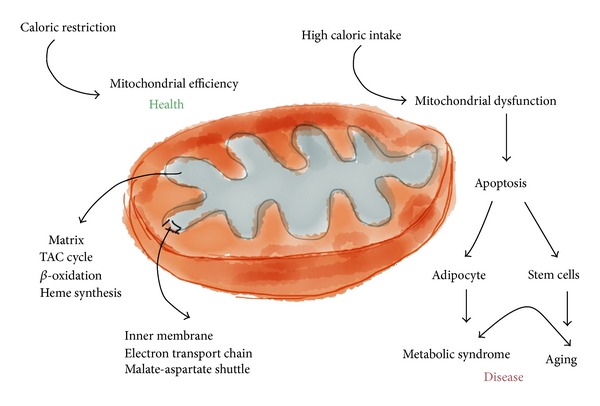
The mitochondrial matrix hosts the mitochondrial metabolic pathways (TAC cycle, *β*-oxidation, and haem synthesis), and the inner membrane contains the electron transport chain complexes and ATP synthase. Exchange carriers such as the malate-aspartate shuttle are also essential. Under caloric restriction, the mitochondrion achieves the highest efficiency, and high caloric intake produces dysfunction and a consequent increase in apoptosis, which promotes metabolic syndrome and age-related diseases.

**Figure 4:**
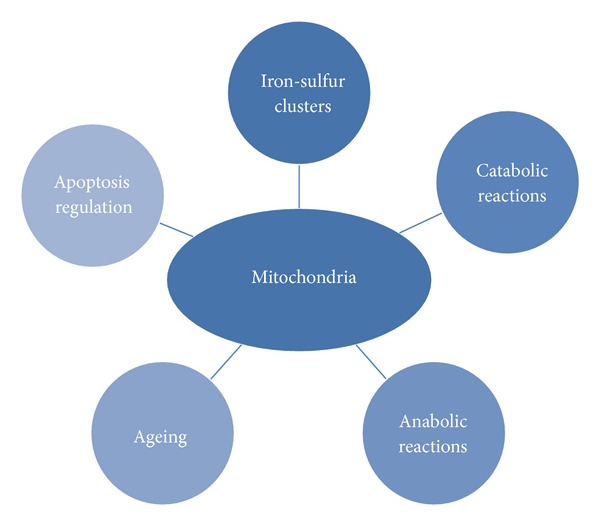
Schematic and abridged representation of the multiple roles of mitochondria in cellular processes that are associated with the pathogenesis of the more prevalent diseases.

Incidentally, the role of genetics in low-energy expenditure and chronic food intake, although potentially significant, remains poorly understood [29, 30]. The genetic-selection hypothesis, which attempts to explain the high prevalence of obesity and diabetes in humans, remains controversial, since the recent abandonment of the “thrifty” gene hypothesis [34–38]. As a result, the roles of oxidative stress, inflammation, mitochondrial dysfunction, nutritional status, and metabolism might be reinforced in hypotheses regarding the pathogenesis of noncommunicable diseases (Figures 3 and 4). 

Inflammation plays a vital role in host defence. Tissue damage, fibrosis, and losses of function occur under chronic inflammatory conditions. Growing evidence links a low-grade, chronic inflammatory state to obesity and its coexisting conditions as well as to noncommunicable diseases [10–16]. This low-grade inflammatory state is aggravated by the recruitment of inflammatory cells, mainly macrophages, to adipose tissue. Inflammatory cell recruitment is likely due to the combined effects of the complex regulatory network of cells and mediators that are designed to resolve inflammatory responses [7]. Anti-inflammatory drugs have shown to reverse insulin resistance and other related conditions that result from circulating cytokines that cause and maintain insulin resistance [19, 23, 39–42]. Therefore, it is likely that inflammation *per se* is a causal factor for noncommunicable diseases rather than an associated risk factor. 

It is also important to highlight that adipose tissue is comprised of multiple types of cells that have intrinsic and important endocrine functions, particularly those mediated by leptin and adiponectin. Recruited and resident macrophages secrete the majority of inflammatory adipokines, specifically tumour necrosis factor *α* (TNF*α*), interleukin-6 (IL-6), and monocyte chemoattractant protein-1 (MCP-1), among others. The major roles of TNF*α* and other inflammatory cytokines in the progression of metabolic complications are likely related to oxidative stress [43, 44]. In adipose tissue macrophages, increased concentrations of saturated free fatty acids (FFAs) stimulate the synthesis of TNF*α* directly through the Toll-like receptor 4 (TLR4) or indirectly through cellular accumulation. Both macrophages and adipocytes possess TLR4 receptors that, upon lipid-dependent activation, induce NF-KB translocation to the nucleus and the subsequent synthesis of TNF*α* and IL-6 [7, 43, 44]. However, recruited macrophages have unique inflammatory properties that are not observed in resident tissue macrophages, and the recruitment of these cells is mainly modulated by MCP-1, the most important molecule of the CC chemokine family [7]. In this setting, the roles and polarisation of adipose tissue macrophages (ATMs) seem established [45]. M1 or “classically activated” ATMs are increased, and M2 or “alternatively activated” ATMs are decreased in the adipose tissues of both obese mice and obese humans, as discussed below [46, 47]. 

It is frequently assumed that, in contrast to hormones, chemokines influence cellular activities in an autocrine or paracrine fashion. However, chemokines may be relevant effectors in chronic systemic inflammation as the confinement of these molecules to well-defined environments is unlikely. Specifically, alterations in plasma MCP-1 concentrations in metabolic disease states, the presence of circulating chemokine reservoirs, recent evidence of novel mechanisms of action, and certain unexplained responses associated with metabolic disturbances suggest that MCP-1 might have a systemic role in metabolic regulation [48–50]. How and when obesity might initiate an inflammatory response remains controversial, but the underlying mechanism likely depends on the activation of the c-Jun N-terminal kinase (JNK) in insulin-sensitive tissues, as JNK is likely the principal mechanism through which inflammatory signals interfere with insulin activity [7]. 

ER stress responses and mitochondrial defects are also linked to the mTOR pathway, discussed below, which is essential for the regulation of numerous processes, including the cell cycle, energy metabolism, the immune response, and autophagy. Therefore, the specific cellular changes associated with metabolic alterations, particularly mitochondrial dysfunction, require further attention. 

## 3. Mitochondria: Bioenergy Couples Metabolism, Oxidation, and Inflammation 

**Figure 5:**
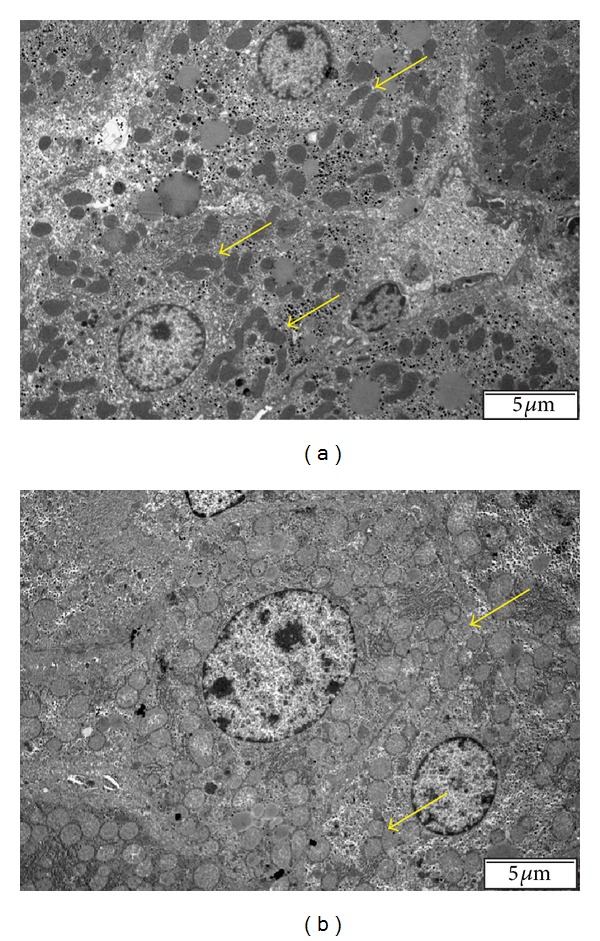
Mitochondrial fusion (a) and fission (b) processes in the liver (arrows). Mitochondrial morphology is basically controlled by metabolism and inflammation, and each change in morphology is mediated by large guanosine triphosphatases of the dynamin family, consistent with a model in which the capacity for oxidative phosphorylation is maximised under stressful conditions.

**Figure 6:**
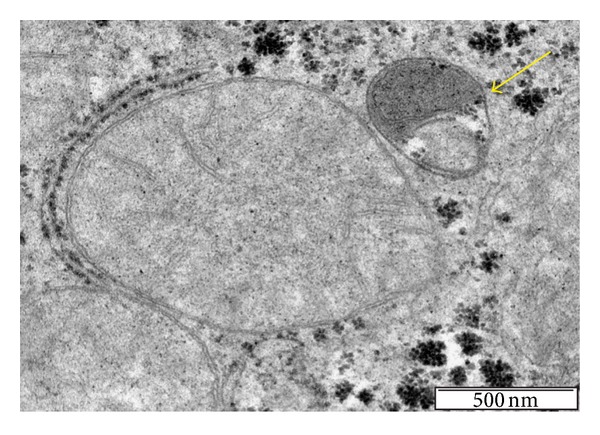
The complete elimination of mitochondria by autophagy (arrow) is a process linked to mitochondrial fission and fusion. Mitochondria also employ quality-control proteases to eliminate damaged molecules through the transcriptional induction of chaperones or the ubiquitin proteasome quality-control pathway.

Mitochondria are essential organelles that, among other functions, supply the cell with ATP through oxidative phosphorylation, synthesise key molecules, and buffer calcium gradients; however, they are also a source of free radicals (Figures 1, 3, and 4). It is not surprising that mitochondrial health is tightly regulated and associated with the homeostasis and aging of the organism. Within these processes, the antagonistic and balanced activities of the fusion and fission machineries constantly provide adequate responses to events caused by inflammation (Figure 5) [23, 50–54]. A shift towards fusion favours the generation of interconnected mitochondria, which contribute to the dissipation and rapid provision of energy. A shift towards fission results in numerous mitochondrial fragments. Apparently, the mixing of the matrix and the inner membrane allows the respiratory machinery components to cooperate most efficiently. Furthermore, fusion maximises ATP synthesis. In quiescent cells, mitochondria are frequently present as numerous morphologically and functionally distinct small spheres or short rods [51, 55, 56]. Upon the exposure of cells to stress, fusion optimises mitochondrial function and plays a beneficial role in the maintenance of long-term bioenergetics capacities. In contrast, the mitochondrial fission machinery contributes to the elimination of irreversibly damaged mitochondria through autophagy [55–58]. This process, also called mitophagy, is extremely important under both physiological and pathological conditions (Figure 6). A detailed discussion of the importance of mitophagy is beyond the scope of this review; however, as an example of its importance, recall that amino acids are not stored in the body but are instead mobilised by proteolysis under conditions such as starvation, reduced physical activity, and disease [59]. Furthermore, intense exercise may modulate hepatic metabolism through similar mechanisms [60]. More recently, the mitochondrial E3 ubiquitin protein ligase 1 (Mul 1) was identified as a key protein that promotes mitophagy and skeletal muscle loss [61]. Mitochondrial fission *per se* triggers organelle dysfunction and muscle loss. The opposite is observed when mitochondrial fission is inhibited. The same authors [61] also demonstrated that the overexpression of Forkhead box O3 (FoxO3) induces mitochondrial disruption via mitophagy. 

 Therefore, it is not surprising that mitochondrial diseases often have an associated metabolic component, and consequently mitochondrial defects are expected in inflammation, aging, and other energy-dependent disturbances [58, 62]. In such disturbances, cellular oxidative damage caused by the generation of reactive oxygen species (ROS) that exceed the natural antioxidant activity is likely an initiating factor in inflammation and aging [63, 64]. Several potential therapeutic approaches are currently available to slow down age-related functional declines [65], including antioxidant treatments [66]; however, the effectiveness of existing antioxidants is likely suboptimal because these antioxidants are not selective for mitochondria [67]. However, recent experiments with a mitochondria-targeted antioxidant have been successful in animal models [67]. Similar assumptions can be made for endothelial cells, in which oxidation and the accompanying inflammation are recognised factors for atherosclerosis. Oxidative stress, which is mainly derived from mitochondrial dysfunction, decreases NO synthesis, contributes to hypertension, upregulates the secretion of adhesion molecules and inflammatory cytokines, and is responsible for the oxidation of low-density lipoproteins [68, 69]. 

Defective mitochondrial function in muscle tissues leads to reduced fatty acid oxidation and the inhibition of glucose transport, indicating that insulin-stimulated glucose transport is reduced. This is a hallmark of insulin resistance and type 2 diabetes. The chronic production of excess ROS and inflammation result in mitochondrial dysfunction potentially inducing lipid accumulation in these tissues and the endless vicious cycle of insulin resistance [70–74]. Mitochondrial ROS have also been related to the increased activity of uncoupling proteins (UCP), which uncouple ATP synthesis from electron transport. UCP activity leads to heat generation without ATP production, and long-term reductions in ATP levels affect cellular insulin signalling. The roles of the UCPs and the metabolically relevant differences between brown and white adipose tissues were reviewed recently [75–77]. 

The mitochondria of obese individuals are different from those of lean individuals. Alterations in mitochondrial morphology, impaired mitochondrial bioenergetics, increased mitochondrial lipid peroxides, decreased ATP content, and mitochondrial dysfunction further increase the risks of developing metabolic complications [78, 79]. In comparison to those of lean individuals, mitochondria in obese individuals have lower energy-generating capacities, less clearly defined inner membranes, and reduced fatty acid oxidation. These differences might promote the development and progression of obesity and might also have therapeutic implications [80, 81]. Impaired mitochondrial function could account for the insulin resistance that is closely associated with increased lipid content in the muscles of patients with type 2 diabetes. Altered mitochondrial function is the major factor that leads to increased muscular lipid accumulation and decreased insulin sensitivity [80, 81]. More recently, a model was created in which the amount of mitochondrial activity in adipocytes and hepatocytes can be altered based on the properties of the mitochondrial protein mitoNEET, which is located at the outer membrane [70]. Despite the prevalence of obesity in this model, mitoNEET overexpression during periods of high caloric intake resulted in systemwide improvements in insulin sensitivity, thereby providing a model of a “metabolically healthy” obese state with minimal tissue lipotoxicity that is similar to the clinically observed condition [82]. Alterations in mitoNEET expression might modulate ROS concentrations and mitochondrial iron transport into the matrix [70, 82, 83]. The mitochondrial fusion protein mitofusin-2 (Mfn-2), another useful protein in studies of mitochondrial dysfunction, regulates cellular metabolism and controls mitochondrial metabolism. In cultured cells, mitochondrial metabolism was activated in Mfn-2 gain-of-function experiments, whereas Mfn-2 loss-of-function reduced glucose oxidation, mitochondrial membrane potential, oxygen consumption, and mitochondrial proton leakage [84]. It is defective in the muscles of obese and type 2 diabetes patients in which mitochondrial size is reduced [71]. 

 Therefore, a detailed characterisation of the proteins involved in mitochondrial fusion and fission and studies of the mechanisms that regulate these two processes are relevant to human pathology and might have a great therapeutic potential to improve metabolism and to decrease the generation of oxidative stress and excessive inflammatory response [85]. 

## 4. Is There a Link between Mitochondria and Nutrient Availability? The Possible Roles of Inflammation and Apoptosis

Apoptosis is another basic process to consider in metabolic diseases. Excess food intake leads to mitochondrial dysfunction and higher apoptotic susceptibility. Mitochondria specialise in energy production and cell killing. Only 13 proteins are encoded by the mitochondrial DNA, a circular molecule of 16 Kb. The remaining necessary proteins are encoded in the nuclear DNA [86]. Mitochondria are composed of outer and inner specialised membranes that define two separate components, the matrix and the intermembrane space [87]. Mitochondria regulate apoptosis in response to cellular stress signals and determine whether cells live or die [88]. Thus, it is conceivable that the availability or ingestion of nutrients could be a main candidate in the regulation of cell death and that mitochondria could have been selected as a nutrient sensor and effector. This could explain the influence of apoptosis-related proteins on mitochondrial respiration [89]. 

**Figure 7:**
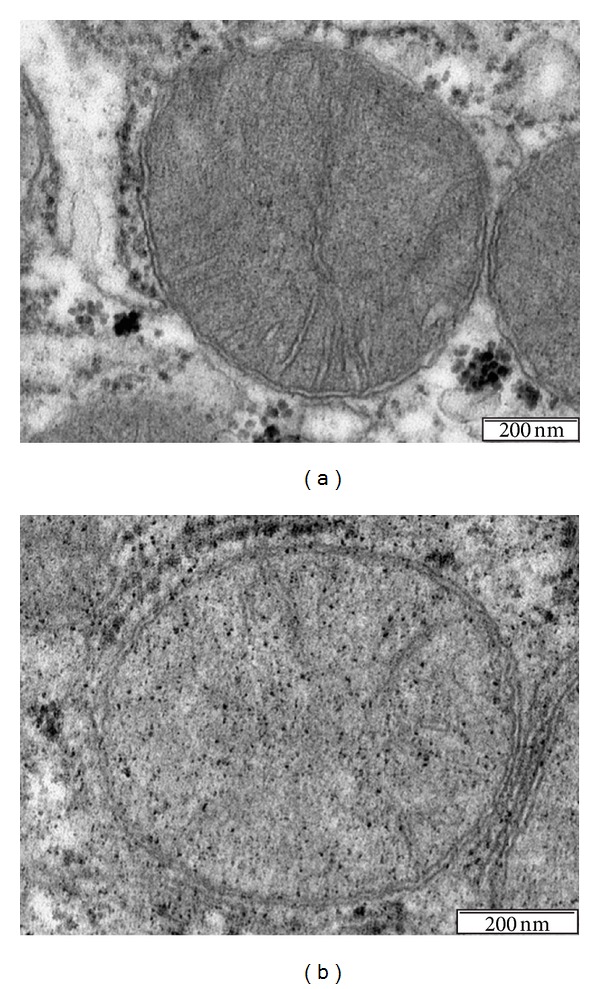
The nutrient availability of food in “natural” conditions for mice is likely low and near the condition known as calorie restriction. In the laboratory, however, mice are usually fed *ad libitum*, and certain biases cannot be discarded. However, mitochondria from mice fed a chow diet (a) display rapid morphological changes when mice are fed with high-fat diets (b).

A common laboratory finding is that the morphology of the mitochondria changes when mice are supplied with a high-fat diet (Figure 7) and that optimal mitochondrial performance is achieved under conditions of calorie restriction. Excess food intake impairs respiratory capacities, likely through mTOR, and increases the susceptibility of the cell to apoptosis and additional stress [90, 91]. Of note, apoptotic protein levels are increased in the adipocytes of obese humans, and the depletion of proapoptotic proteins protects against liver steatosis and insulin resistance in mice fed a high-fat, high-cholesterol diet [92]. These conditions are relevant to the development of metabolic syndrome, as nutritional imbalances in Western diets lead to mitochondrial dysfunction and higher susceptibilities to inflammation, apoptosis, and aging [22]. 

## 5. AMP-Activated Protein Kinase (AMPK) Not Only Influences Metabolism in Adipocytes but Also Suppresses the Proinflammatory Environment

**Figure 8:**
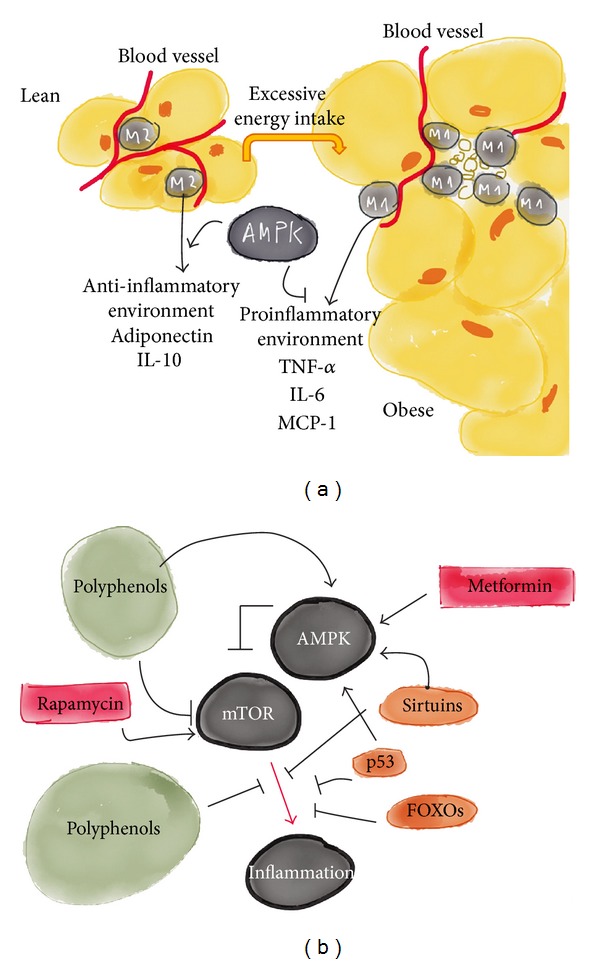
Activation of AMPK in macrophages promotes the switch from a proinflammatory to an anti-inflammatory phenotype by inducing a shift from glycolysis towards mitochondrial oxidative metabolism. In obesity, there may be a shift towards proinflammatory states, whereas in dietary restriction the balance may shift towards anti-inflammatory phenotypes through the activation of AMPK (a). The activation of AMPK implies the inhibition of mTOR, and several compounds are known to regulate this pathway (b). The inhibition of mTOR extends lifespan in model organisms and confers protection against a growing list of age-related pathologies. Several characterised inhibitors are already clinically approved, and others are under development.

AMPK has anti-inflammatory actions that are independent of its effects on glucose and lipid metabolism [93]. The action of AMPK is not necessarily identical in all tissues. In adipose tissues, the role of AMPK is largely unknown because laboratory techniques to explore the action of this kinase in terminally differentiated adipocytes have not been fully established. Several agents have been used to activate AMPK experimentally, including AICAR (5′-aminoimidazole-4-carboxamide ribonucleoside), metformin, rosiglitazone, resveratrol and other polyphenols, statins, and several adipocytokines. In adipocytes, AMPK appears to increase the insulin-stimulated uptake of glucose, likely by increasing the expression of GLUT4, yet inhibits glucose metabolism [94]. Studies of the effects of AMPK on lipolysis in adipocytes have been controversial; some authors have reported an antilipolytic effect, while others have suggested that AMPK stimulates lipolysis [95, 96]. However, the activation of AMPK by metformin in human adipose tissues increases the phosphorylation of acetyl-CoA carboxylase (ACC) and decreases the expression of lipogenic genes, leading to reductions in malonyl-CoA, which is the precursor for fatty acid synthesis; malonyl-CoA also regulates fatty acid oxidation through the inhibition of carnitine palmitoyl-transferase 1, the rate-limiting enzyme for fatty acid entry into the mitochondria [97, 98]. Adipose tissue secretes adipocytokines, which influence metabolic and inflammatory pathways through the recruitment of macrophages and the consequent transition from the M2 state to M1 [7, 41]. These actions contribute to the development of disease (Figure 8). Conversely, adiponectin has been reported to induce adipose macrophages to switch to the anti-inflammatory M2 state [99]. AMPK is anti-inflammatory, as it inhibits the synthesis of proinflammatory cytokines and promotes the expression of IL-10 in macrophages; adiponectin and leptin levels may also be regulated by AMPK [100] (Figure 8). Finally, brown adipocytes contain high numbers of mitochondria that express UCP1, which permit thermogenesis. Exposure to cold temperatures stimulates AMPK and may play a role in the differentiation of fatty oxidising brown adipose tissue, thus leading to greater energy expenditure [101]. Therefore, we hypothesise that the chronic manipulation of the AMPK/mechanistic target of rapamycin (mTOR) pathway might represent a therapeutic approach for preventing noncommunicable diseases (Figure 8). Metformin, along with salicylate, polyphenols, and rapamycin, has a long history of safe and effective use, but other modulators are currently under development and will likely permit the design of tissue-specific activators of this pathway. 

## 6. Metformin and/or Rapamycin and Plant-Derived Polyphenols: An Apparent Treatment of Choice for Metabolic Syndrome and Obesity-Related Complications? 

The first therapeutic approaches to metabolic disturbances are reduced caloric ingestion and increased physical activity. The effects are based mainly on weight reduction, but usefulness in other common complications remains incompletely explored [102]. Bariatric surgery is also effective, even in “metabolically healthy” patients [103, 104]. The effectiveness of surgery for the treatment of metabolic disturbances is surprisingly higher than expected, and mechanisms associated with surgical effects are not completely understood. 

Insulin resistance and mitochondrial dysfunction appear to be the most significant alternative therapeutic targets. Metabolic abnormalities are associated with inflammation. Normally, glycolysis yields pyruvate, which is further oxidised in the mitochondria. When oxygen becomes limiting, mitochondrial oxidative metabolism is restricted. The induction of an inflammatory response is an energy-intensive process, and the involved cells rapidly switch from resting to highly active states. This is observed in diseases such as cancer, atherosclerosis, or autoimmune diseases, and mechanistic insights suggest the common involvement of the transcription factor hypoxia-inducible factor 1*α*, AMPK, and the mTOR pathway. In addition, the activation of sirtuins, which act as NAD+ sensors that connect nutrition and metabolism to chromatin structure, is anti-inflammatory [105] (Figure 8). 

The use of metformin, an AMPK activator used extensively to treat type 2 diabetes, has been indicated for other metabolic conditions based on the rationale that insulin-sensitising agents might be effective [106], and the mode of action of metformin has guided our own experiments on cancer, aging, and viral infection [65, 107, 108]. We have shown that the beneficial effects of this biguanide class drug, which was initially obtained from *Galega officinalis*, are universal in patients with metabolic complications and negligible in patients without such complications. The primary effect is thought to be the suppression of hepatic glucose production and hepatic lipogenesis [109]. Metformin activates AMPK in hepatocytes, resulting in the phosphorylation and inactivation of ACA, a rate-limiting enzyme in lipogenesis [110], and theoretically might be useful and safe in the treatment of NAFLD [111]. Surprisingly, the beneficial clinical effects seem to be limited, despite the effects of metformin on insulin resistance, most likely because long-term treatment is an absolute requirement for the prevention of progressive disease. Our own current experiments in animal models suggest new insights into this phenomenon. Metformin activates AMPK, but AMPK deficiency does not abolish the effects of metformin on hepatic glucose production, indicating that the role of AMPK is dispensable, as indicated previously [112]. This suggests that the overall effect of metformin is mediated through actions on mitochondrial function through decreases in the hepatic energy state and intracellular ATP content. Other studies suggest that metformin inhibits Complex I of the mitochondrial respiratory chain, but the exact mechanisms and pathways involved are unclear [113]. Sirtuin 3 (SIRT 3), a member of the family of nicotinamide adenine dinucleotide (NAD+) dependent deacetylase proteins, is a crucial regulator of mitochondrial function that controls the global acetylation of the organelle (all sirtuins regulate energy production and the cell cycle; Figure 8). SIRT3 induces the activity of Complex I and promotes oxidative phosphorylation. In SIRT3 knockout mice, mitochondrial proteins are hyperacetylated, and cellular ATP levels are reduced, effects that are aggravated by fasting [114]. As a complement, peroxisome proliferator-activated receptor gamma coactivator 1-alpha induces the expression of SIRT3 in the liver [115]. Therefore, mitochondrial function appears to be the key target of metformin; reductions in ATP production may mediate the hepatic and antihyperglycemic actions of the drug and downregulate SIRT3 expression [116]. However, metformin distinctively regulates the expression of different sirtuin family members [117, 118]. In summary, metformin acts against both insulin resistance and mitochondrial dysfunction and is currently an attractive candidate agent of choice in the management of metabolic disorders. We have recently reviewed this complex scenario and found the following: (1) the unique ability of metformin to activate AMPK while leading to the increased utilisation of energy occurs because metformin inhibits AMP deaminase; and (2) in metabolic tissues, metformin can inhibit cell growth by functionally mimicking the effects of a multitargeted antifolate [119]. 

Based on these and other findings, we have also demonstrated that plant-derived phenolic compounds interact with numerous targets and multiple deregulated signalling pathways that may be useful in the management of metabolic conditions [120–123]. The proposed mechanisms are direct antioxidant activity, attenuation of endoplasmic reticulum stress, blockade of proinflammatory cytokines, and blockade of transcription factors related to metabolic diseases [120]. Most polyphenols modulate oxidative stress and inflammatory responses through relevant actions in the process of macrophage recruitment. Interactions between the chemokine/cytokine network and bioenergetics, likely through the mTOR pathway, may also represent potential mechanisms for the prevention of metabolic disturbances [121]. Moreover, polyphenols attenuate the metabolic effects of high-fat, high-cholesterol diets when administered continuously at high doses, and we have described beneficial actions associated with the expression of selected microRNAs [122]. 

Inflammation lies at the heart of many diseases because the entire body is under metabolic stress, which induces symptoms and causes morbidity. Targeting altered metabolic pathways in inflammation may enhance our understanding of disease pathogenesis and point the way to new therapies. As mentioned, metformin, polyphenols, AICAR, salicylates, and corticoids all activate the AMPK/mTOR pathway. New compounds such as A-769662 are under scrutiny. Finally, rapamycin, which is also known as sirolimus and was first isolated from *Streptomyces hygroscopicus*, and several derivative compounds, including everolimus, temsirolimus, ridaforolimus, umirolimus, and zotarolimus, have been approved for a variety of uses, including posttransplantation therapy, the prevention of restenosis following angioplasty, and as a treatment for certain forms of cancer. Drugs that inhibit the mTOR pathway could one day be used widely to slow aging and reduce age-related pathologies in humans [124]. The development of chemical inhibitors of mTOR, as well as drugs that target other components of the mTOR pathway, promises to aid research greatly while also providing drugs with potential therapeutic value. 

## 7. Perspectives and Implications

Obesity, metabolic alterations, and age-related diseases are complex conditions that require a multifaceted approach that includes action on both the chemokine network and energy metabolism [123, 125]. The underlying mechanisms are far from being understood [126] although the association between obesity and insulin resistance seems to be well substantiated. However, obesity is not distributed normally throughout the population, and type 2 diabetes mellitus is not associated closely with increased body weight; also, the relationship with noncommunicable diseases is not straightforward. A working hypothesis is that adipose tissue has a limited maximum capacity to increase in mass. Once the adipose tissue has reached the expansion limit, fat is deposited in the liver and muscle tissues and causes insulin resistance. This process is also associated with the activation of macrophages, oxidative stress, and inflammation which produce cytokines that have negative effects on insulin sensitivity, induce the secretion of adipokines that cause insulin resistance, and suppress those that promote insulin sensitivity. However, a host of other mechanisms must be involved because metabolic responses are different among patients with maximum adipose tissue expansion. A more popular and recent hypothesis suggests a differential effect of lipophagy, which implies a tissue-selective autophagy with cellular consequences from the mobilisation of intracellular lipids. Defective lipophagy is linked to fatty liver tissues and obesity and might be the basis for age-related metabolic syndrome [127]. Increased adipose tissue autophagy may be responsible for more efficient storage. Autophagy also affects metabolism, oxidation, and proinflammatory cytokine production. Very recent evidence suggests that autophagy is increased in the adipose tissues of obese patients [128]. Inexpensive and well-tolerated molecules such as chloroquine, metformin, and polyphenols already exist and could be used to fine-tune the metabolic alterations derived from an excess of energy and, more specifically, to modulate autophagy in the liver. Whether these therapies will dampen the genetic expression of factors that affect the development of noncommunicable diseases remains to be ascertained.
